# Facial and Dentoalveolar Changes Following Orthodontic Treatment of Bimaxillary Protrusion in Two Sisters

**DOI:** 10.7759/cureus.117207

**Published:** 2026-09-30

**Authors:** Tonamou François, Meriem Bellamine, Amadou Oury Diallo, Chaymae Chafik, Ihsane Benyahya

**Affiliations:** 1 Orthodontics and Dentofacial Orthopedics, Mohammed VI University of Health Sciences, Casablanca, MAR; 2 Dentofacial Orthopedics, Mohammed VI University of Health Sciences, Casablanca, MAR; 3 Surgical Odontology, Faculty Mohammed VI of Dental Medicine, Mohammed VI University of Health Sciences, Mohammed VI Foundation of Health and Sciences, Casablanca, MAR

**Keywords:** bimaxillary dentoalveolar protrusion, dentoalveolar response, orthodontic treatment, premolar extraction, soft-tissue response

## Abstract

Bimaxillary dentoalveolar protrusion is characterized by excessive labial inclination and protrusion of the maxillary and mandibular incisors and is frequently associated with lip protrusion, lip incompetence, and increased facial profile convexity. Orthodontic treatment, particularly when combined with premolar extractions, aims to retract and reposition the anterior dentoalveolar segments, thereby improving dentofacial relationships and the soft-tissue profile.

The soft-tissue response to dentoalveolar changes, however, may vary according to individual anatomical characteristics, growth status, and the timing of orthodontic intervention. We report the cases of two sisters presenting with bimaxillary dentoalveolar protrusion and broadly similar pretreatment dentofacial characteristics. These similarities included a comparable skeletal pattern, maxillary and mandibular incisor protrusion, and proclination, as well as similar soft-tissue features characterized by lip protrusion, lip incompetence, and increased facial profile convexity. Despite these similarities, the two patients differed substantially in age and growth status, providing an opportunity to descriptively compare their dentoalveolar and soft-tissue responses to orthodontic treatment performed at different stages of dentofacial development.

The younger sister, aged 12 years, was still undergoing craniofacial growth and was treated during the transition to permanent dentition. At the initial examination, some primary teeth were still present. These teeth were allowed to exfoliate physiologically, after which the first permanent premolars were extracted as part of the orthodontic treatment plan. The older sister, aged 23 years, presented with a fully established permanent dentition and completed craniofacial growth and was treated with extraction of the second premolars. Both patients were treated by the same orthodontist.

Changes in incisor position and the perioral soft-tissue profile were assessed clinically and cephalometrically before and after orthodontic treatment, with long-term follow-up. In both patients, treatment resulted in retraction and uprighting of the incisors, accompanied by improvement in the soft-tissue profile and lip relationship. In Case 1, mandibular incisor uprighting was confirmed by a decrease in the incisor mandibular plane angle from 104.3° before treatment to 100.9° after treatment. Improved lip competence and reduced visible mentalis muscle activity during lip closure were also observed clinically. These soft-tissue findings were based on clinical examination and photographic assessment and were not evaluated using a standardized quantitative protocol.

The comparison of these two cases suggests that the timing of orthodontic intervention and the patient's growth status may be associated with differences in the dentoalveolar and soft-tissue response to the treatment of bimaxillary dentoalveolar protrusion. Nevertheless, these findings are exploratory and should be interpreted cautiously. Given the descriptive nature of this two-case comparison and the differences in treatment protocol, particularly the premolars selected for extraction, no causal relationship between growth status, treatment timing, and treatment response can be established.

## Introduction

Contemporary orthodontic treatment goals extend beyond the correction of malocclusion and the establishment of optimal dentoskeletal relationships to include facial esthetics and soft-tissue harmony. In particular, the relationship between the lips and the underlying dentoalveolar structures is an important consideration in treatment planning and outcome assessment. The position and inclination of the maxillary and mandibular incisors contribute to lip support and facial profile morphology; consequently, orthodontic repositioning of the anterior teeth may induce clinically relevant changes in the perioral soft tissues.

Bimaxillary dentoalveolar protrusion is characterized by excessive proclination and protrusion of the maxillary and mandibular incisors and may be associated with lip protrusion, lip incompetence, increased facial profile convexity, and mentalis muscle activity during attempted lip closure. When reduction of anterior dental and soft-tissue protrusion is a treatment objective, premolar extraction may provide the space required for retraction and controlled repositioning of the anterior dentoalveolar segments. Previous studies have reported that extraction-based orthodontic treatment may result in lip retraction and measurable changes in facial soft-tissue profile parameters [1-3]. Furthermore, systematic reviews evaluating anterior retraction in bimaxillary dentoalveolar protrusion have shown significant improvement in perioral aesthetics alongside dental uprighting [4].

However, the soft-tissue response to incisor retraction is neither uniform among patients nor directly proportional to the magnitude of tooth movement. Previous studies and systematic reviews have demonstrated substantial interindividual variability, suggesting that soft-tissue changes are influenced by multiple patient- and treatment-related factors. These include the magnitude and direction of incisor movement, extraction pattern, anchorage requirements, and space-closure mechanics, as well as baseline soft-tissue characteristics such as lip thickness, lip strain, and initial lip position [2,5,6]. A recent meta-analysis confirmed significant lip retraction following extraction-based orthodontic treatment but also reported considerable heterogeneity among studies, limiting the ability to reliably predict the magnitude of soft-tissue changes in individual patients [5].

The relationship between incisor retraction and the resulting soft-tissue response therefore appears to be complex and patient-specific. In adult patients, recent evidence suggests that baseline vermilion thickness may influence the magnitude of lip retraction following premolar extraction [7]. These findings emphasize that changes in facial profile cannot be predicted solely from the amount of incisor movement and should instead be interpreted within the broader context of the patient's initial dentofacial morphology, soft-tissue characteristics, and treatment protocol.

Growth status may represent an additional factor to consider when interpreting treatment-related dentofacial changes. In growing patients, orthodontic dentoalveolar changes occur concurrently with ongoing craniofacial growth and maturation, whereas after growth completion, this concurrent influence of active craniofacial growth is substantially reduced. Consequently, comparison of patients treated at different developmental stages may provide descriptive insight into dentoalveolar and soft-tissue changes occurring in different growth contexts. However, growth-related changes cannot be isolated from treatment effects in an uncontrolled case comparison, and differences in baseline craniofacial morphology, dental characteristics, extraction protocol, and treatment mechanics may independently contribute to the observed response. Such comparisons should therefore be interpreted cautiously and should not be considered evidence of a causal effect of growth status or treatment timing.

Against this background, the present case series describes the orthodontic management of two sisters with a similar clinical presentation of bimaxillary dentoalveolar protrusion who were treated at different stages of craniofacial development. Despite their shared clinical presentation, the patients differed in age, growth status, baseline sagittal and vertical skeletal relationships, severity of mandibular crowding, and extraction protocol. The younger patient was treated during active craniofacial growth with extraction of the four first premolars, whereas the older patient was treated after growth completion with extraction of the four second premolars. The aim of this case series was to describe and compare the dentoalveolar and facial soft-tissue changes observed following orthodontic treatment, with particular emphasis on incisor repositioning, lip position and competence, and facial profile changes. The comparison is descriptive and exploratory; the observed differences are interpreted in the context of growth status, baseline dentoskeletal characteristics, and treatment-related factors and are not intended to establish a causal relationship between treatment timing and treatment response.

## Case presentation

Case 1: diagnosis

A 12-year-old female patient presented to our department with a chief complaint related to facial and dental esthetics. She expressed concern regarding the protrusive appearance of her maxillary and mandibular anterior teeth, which she perceived as adversely affecting her facial profile.

Extraoral examination revealed an oval facial form (Figure 1A). Frontal assessment showed an increased lower facial third relative to the middle facial third (Figure 1B). Profile examination revealed a convex facial profile associated with lip incompetence and visible mentalis muscle activity during attempted lip closure (Figure 1B). The nasolabial angle was approximately 90°. Intraoral examination revealed a bilateral class I molar relationship. The canine relationship was Class II on the right side, whereas it could not be assessed on the left because the left maxillary permanent canine had not yet erupted (Figures 1D, 1F). The maxillary arch was narrow and V-shaped, with a high-arched palate. At treatment initiation, the right maxillary second primary molar and the left maxillary primary canine were still retained in the arch (Figure 1G). Their permanent successors, the right maxillary second premolar and the left maxillary permanent canine, respectively, were unerupted and in the process of eruption. The mandibular arch exhibited an ovoid form with mild anterior dental crowding (Figure 1H).

**Figure 1:**
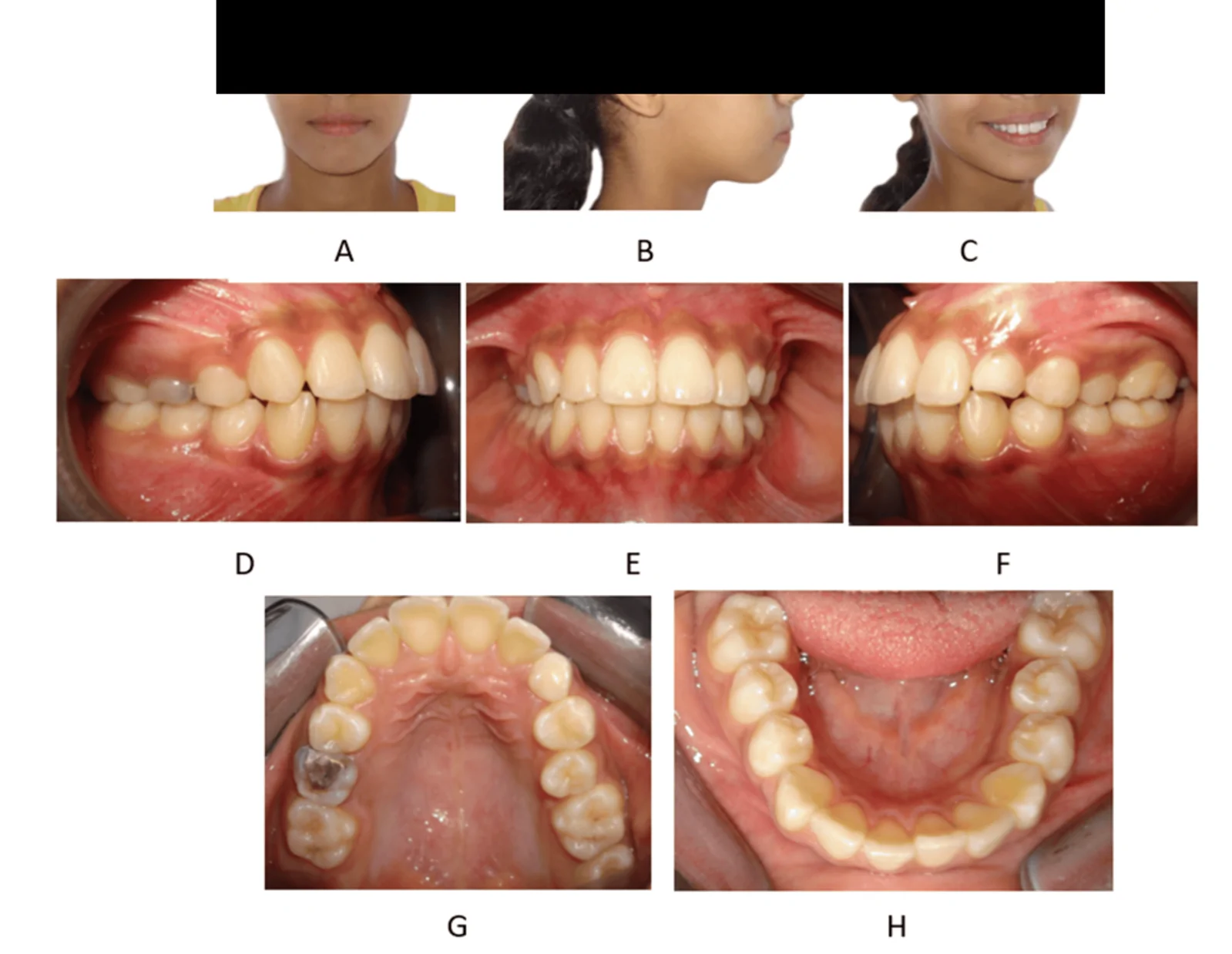
Pretreatment extraoral and intraoral photographs: frontal facial view (A), profile view (B), three-quarter smiling view (C), right lateral intraoral view (D), frontal intraoral view (E), left lateral intraoral view (F), maxillary occlusal view (G), and mandibular occlusal view (H).

The pretreatment panoramic radiograph (Figure 2A) demonstrated a developing permanent dentition, consistent with the patient's transitional stage of dental development. The right maxillary second premolar and the left maxillary permanent canine were unerupted and in the process of eruption beneath their retained primary predecessors. The retained right maxillary second primary molar and left maxillary primary canine showed advanced physiological root resorption, consistent with the ongoing transition to the permanent dentition. All four third molar germs were also visible at an early stage of intraosseous development. The retained primary teeth subsequently exfoliated physiologically before the planned therapeutic extraction of the first permanent premolars. Pretreatment cephalometric analysis (Figure 2B, Table 1) revealed a skeletal class II sagittal relationship, with an ANB (A point-nasion-B point) angle of 7.5°. Vertical skeletal assessment showed an increased SN-GoGn angle of 37.5° and a Frankfort mandibular plane angle (FMA) of 29.3°, indicating a hyperdivergent skeletal pattern. Dentoalveolar analysis demonstrated marked proclination of both the maxillary and mandibular incisors, with a reduced interincisal angle of 116.3°. Overall, the clinical and cephalometric findings were consistent with a diagnosis of bimaxillary dentoalveolar protrusion associated with a skeletal class II relationship and a hyperdivergent growth pattern.

**Figure 2:**
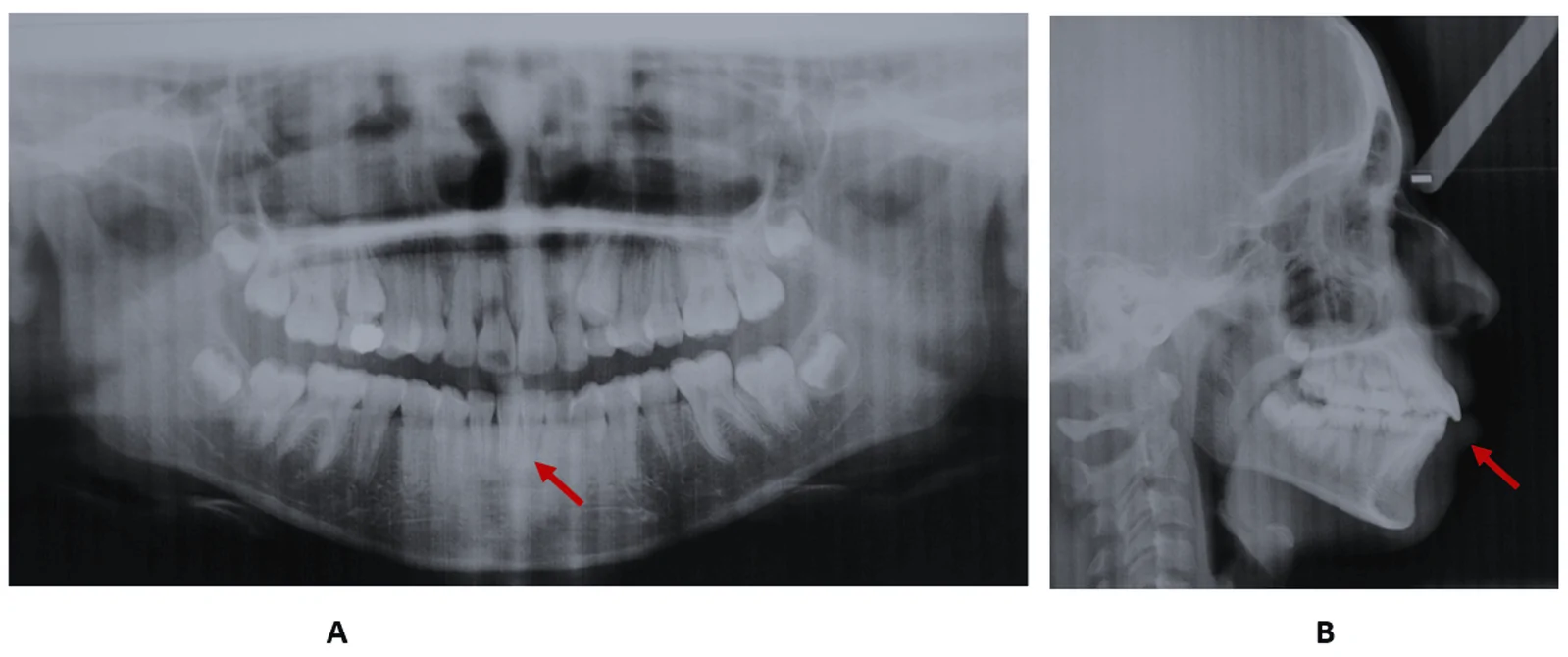
Pretreatment panoramic radiograph showing reduced visualization of the incisor roots due to their position outside the focal trough, consistent with marked incisor proclination (arrow). (B) Pretreatment lateral cephalometric radiograph illustrating the associated dentoalveolar protrusion and soft-tissue profile (arrow).

**Table 1:** Pretreatment and posttreatment cephalometric measurements. SNA, sella–nasion–A point angle; SNB, sella–nasion–B point angle; ANB, A point–nasion–B point angle; AoBo, Wits appraisal (distance between points AO and BO); U1–NA, maxillary central incisor long axis to nasion–A point line; L1–NB, mandibular central incisor long axis to nasion–B point line; interincisal angle, angle between the long axes of the maxillary and mandibular central incisors; Pog–NB, perpendicular distance from pogonion to the nasion–B point line; occlusal plane–SN, angle between the occlusal plane and the sella–nasion plane; SN–GoGn, angle between the sella–nasion plane and the mandibular plane (gonion–gnathion); FMA, Frankfort mandibular plane angle; FMIA, Frankfort mandibular incisor angle; IMPA, incisor mandibular plane angle

Parameter	Norm	Pretreatment	Posttreatment
SNA (°)	82°	87.1°	85.3°
SNB (°)	80°	79.6°	79.2°
ANB (°)	2°	7.5°	6.1°
AoBo (mm)	−2 to +2 mm	2.3 mm	2.1 mm
U1–NA (°)	22°	24.7°	22.0°
U1–NA (mm)	4 mm	4.7 mm	3.5 mm
L1–NB (°)	25°	30.3°	26.8°
L1–NB (mm)	4 mm	6.0 mm	5.6 mm
Interincisal angle (°)	131°	116.3°	127.3°
Pog–NB (mm)	-	1.0 mm	1.0 mm
Occlusal plane–SN (°)	14°	20.4°	19.5°
SN–GoGn (°)	32°	37.5°	34.1°
FMA (°)	25 ± 3°	29.3°	26.3°
FMIA (°)	67 ± 3°	46.4°	52.8°
IMPA (°)	88 ± 3°	104.3°	100.9°

Treatment objectives

The treatment objectives were to address the patient’s chief complaint by reducing the bimaxillary dentoalveolar protrusion and improving lip competence and facial profile harmony. Additional objectives included achieving bilateral class I canine relationships while maintaining the existing class I molar relationships, resolving mandibular anterior crowding, and establishing a stable, functional, and well-intercuspal occlusion. From a soft-tissue perspective, treatment aimed to reduce mentalis muscle hyperactivity during lip closure while maintaining a harmonious nasolabial angle.

Treatment plan and progress

An extraction-based orthodontic treatment plan involving the four first premolars was selected to provide sufficient space for anterior tooth retraction and resolution of mandibular crowding. Given the patient’s age and ongoing craniofacial growth, residual growth potential and dental development were considered during treatment planning.

A maximum anchorage strategy based on Tweed-Merrifield biomechanical principles was adopted. Posterior anchorage preparation, particularly at the molar level, was performed according to the Tweed technique before anterior retraction. The objective was to reinforce the posterior anchorage unit, minimize undesirable mesial movement of the posterior teeth, and preserve the extraction spaces predominantly for retraction of the anterior dentoalveolar segments.

Following physiological exfoliation of the retained primary teeth and eruption of their permanent successors to an appropriate stage, the four first premolars were extracted and fixed appliance therapy was initiated. Alignment, leveling, and derotation were performed using an initial 0.014-inch nickel-titanium (NiTi) archwire. Archwire dimensions were progressively increased to complete alignment and leveling of both dental arches and to prepare the dentition for anchorage preparation and subsequent space closure. Controlling incisor inclination and torque during retraction mechanics represents a critical determinant of post-treatment stability and soft-tissue harmony [8].

Extraction-space closure was subsequently performed through progressive canine retraction followed by retraction of the anterior segments while maintaining maximum posterior anchorage. Incisor inclination and torque were controlled according to Tweed-Merrifield biomechanical principles, with progressive transition to rectangular archwires to provide three-dimensional control of the anterior teeth during retraction. These mechanics were intended to limit uncontrolled lingual crown tipping and promote controlled retraction and uprighting of the incisors.

Particular attention was also given to vertical control because of the patient’s initial hyperdivergent skeletal pattern. Posterior anchorage preparation and the mechanics used during space closure were designed to limit undesirable posterior extrusion and clockwise mandibular rotation while maintaining vertical control during anterior retraction.

During the finishing and detailing phase, 0.019 × 0.025-inch stainless-steel archwires were used to optimize arch coordination, root positioning, torque control, and occlusal relationships (Figures 3A-3C). Intermaxillary elastics (6 oz) were subsequently used to refine the interarch relationships and achieve satisfactory intercuspation (Figures 3D-3F).

**Figure 3:**
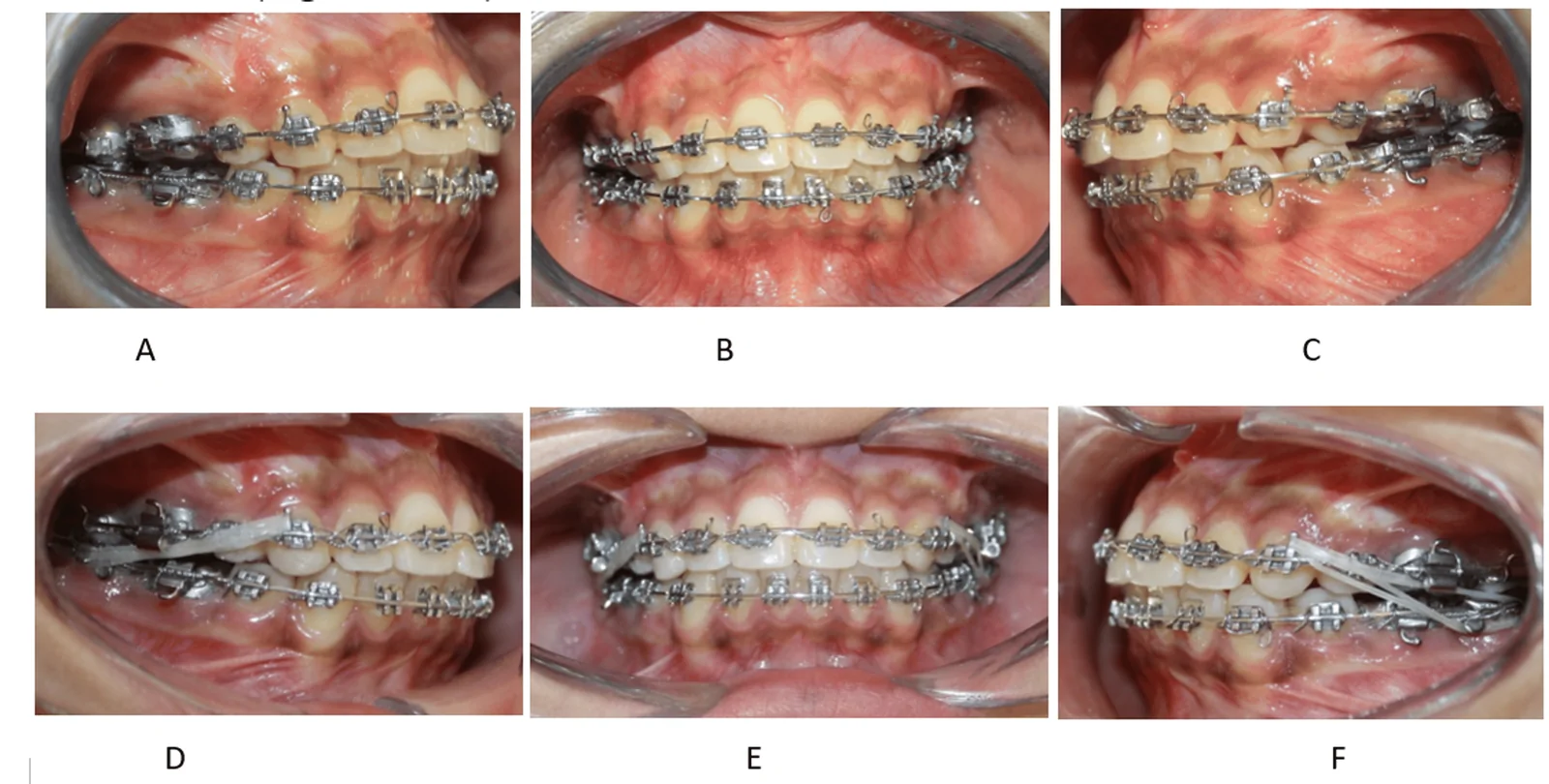
Intraoral photographs during active orthodontic treatment.

Treatment outcomes

Posttreatment extraoral and intraoral photographs showed a clinically observed improvement in facial profile and lip competence, together with the achievement of bilateral class I canine relationships while maintaining the pre-existing class I molar relationships (Figures 4A-4G). The changes in lip competence and facial profile were assessed clinically and photographically rather than through a standardized quantitative soft-tissue assessment.

**Figure 4:**
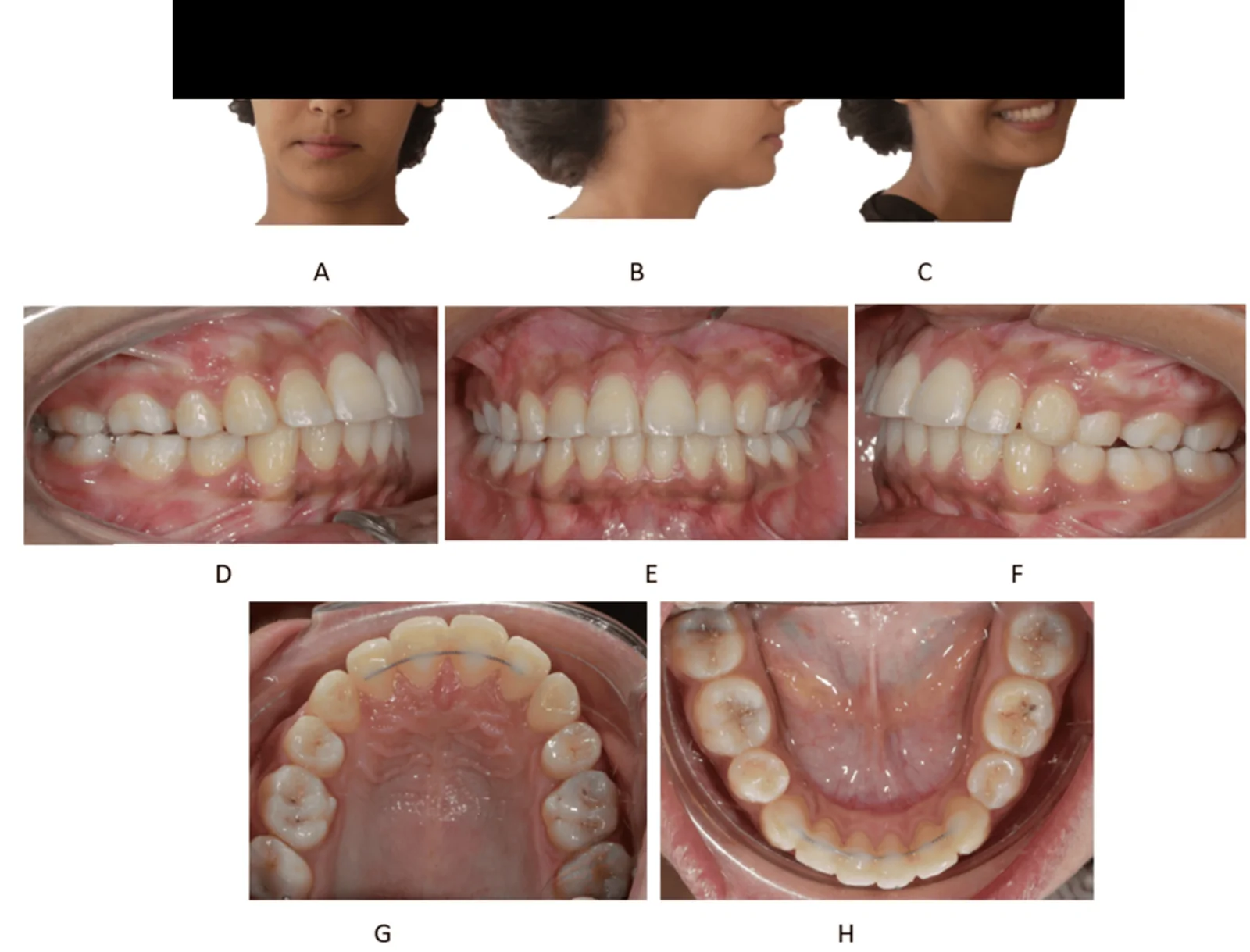
Posttreatment extraoral and intraoral photographs: frontal facial view (A), profile view (B), three-quarter smiling view (C), right lateral intraoral view (D), frontal intraoral view (E), left lateral intraoral view (F), maxillary occlusal view (G), and mandibular occlusal view (H).

The posttreatment panoramic radiograph showed satisfactory root parallelism and overall dental alignment. The third molars remained under development at this stage (Figure 5A). The posttreatment lateral cephalometric radiograph showed clinically apparent changes in the perioral soft-tissue profile and lip relationship (Figure 5B). As no standardized quantitative soft-tissue measurements were performed, these changes were interpreted qualitatively from the clinical photographs and cephalometric records.

**Figure 5:**
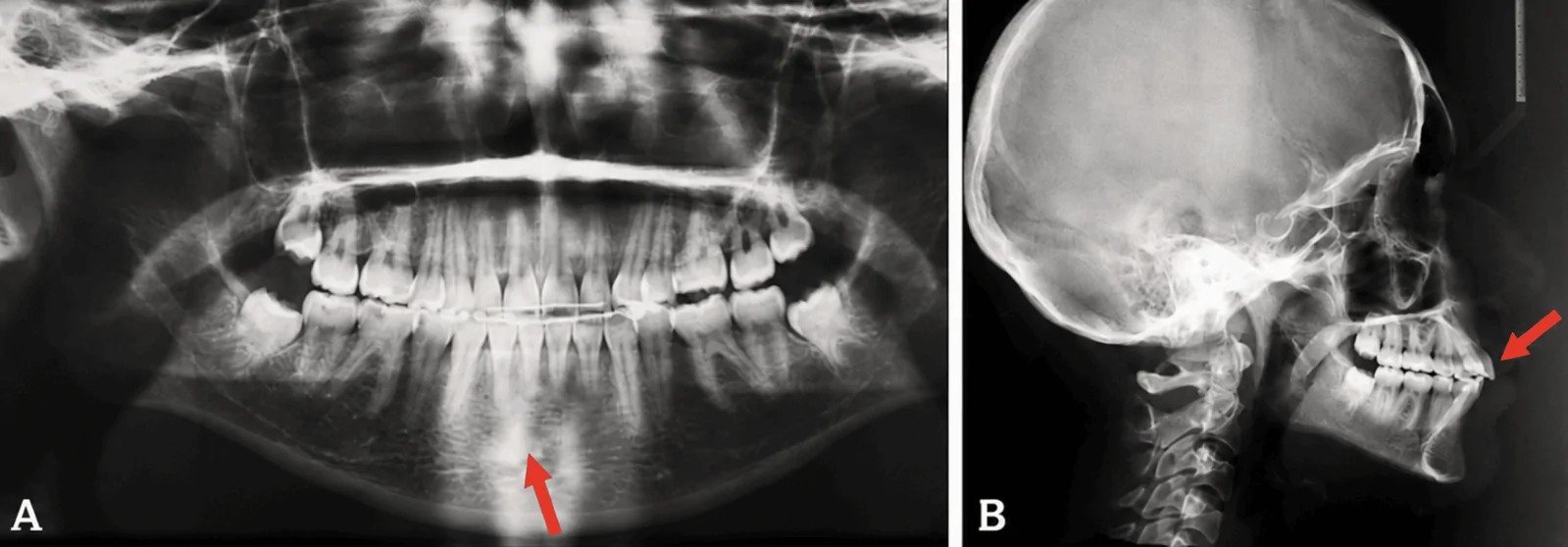
Posttreatment radiographic records. (A) Panoramic radiograph showing incomplete visualization of the mandibular incisor roots, likely related to their slightly proclined position outside the focal trough (arrow). (B) Lateral cephalometric radiograph showing appropriate torque control of the maxillary and mandibular incisors following retraction and uprighting, associated with a straighter and more harmonious soft-tissue profile (arrow).

Posttreatment cephalometric analysis showed a reduction in the ANB angle from 7.5° to 6.1°. Dentoalveolar changes were characterized by retraction and reduced proclination of the maxillary and mandibular incisors, with an increase in the interincisal angle from 116.3° to 127.3°. Mandibular incisor inclination decreased, as demonstrated by a reduction in L1-NB from 30.3° to 26.8° and in incisor mandibular plane angle (IMPA) from 104.3° to 100.9°. Based on the Tweed triangle, FMIA increased from 46.4° to 52.8°. The previously reported reversal of the pretreatment and posttreatment IMPA values was due to a transcription error and has been corrected in Table 1.

In the vertical dimension, the SN-GoGn angle decreased from 37.5° to 34.1°, while the FMA decreased from 29.3° to 26.3°. However, because the patient was undergoing active craniofacial growth during treatment, changes in sagittal and vertical skeletal measurements, including ANB, SN-GoGn, and FMA, cannot be attributed exclusively to orthodontic treatment and should be interpreted as reflecting the combined effects of physiological craniofacial growth and orthodontic treatment.

Pretreatment and posttreatment cephalometric superimposition demonstrated predominantly dentoalveolar changes, particularly retraction and uprighting of the maxillary and mandibular incisors (Figure 6). These dental changes were accompanied by a clinically observed improvement in lip competence and facial profile. Because quantitative soft-tissue measurements were not performed, these soft-tissue changes should be regarded as qualitative clinical observations rather than quantitatively established treatment effects. Furthermore, the overall cephalometric changes observed in this growing patient represent the combined influence of orthodontic treatment and ongoing craniofacial growth.

**Figure 6:**
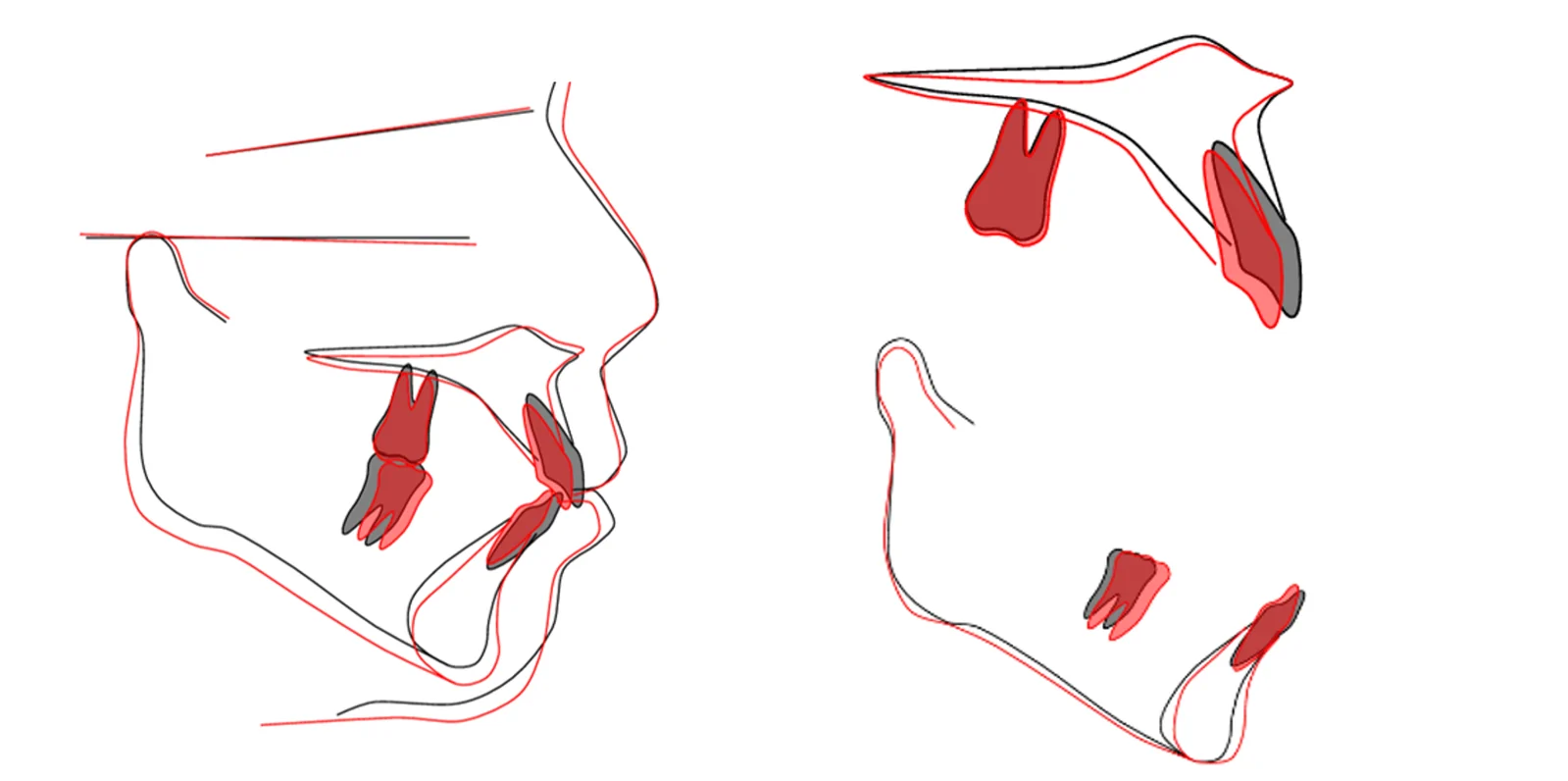
Pretreatment (black) and posttreatment (red) cephalometric superimposition illustrating the skeletal, dentoalveolar, and soft-tissue changes achieved following orthodontic treatment. The image was created using Dental Imaging Studio software (version 1.23; Panoramic Corp).

Case 2: diagnosis

The older sister, aged 23 years, presented to our department with a chief complaint similar to that of her younger sister, primarily related to the protrusive appearance of her anterior teeth and its unfavorable effect on facial esthetics.

Extraoral examination revealed a symmetrical, oval facial form (Figure 7A). Profile assessment showed increased facial convexity associated with lip incompetence at rest and compensatory mentalis muscle hyperactivity during attempted lip closure (Figure 7B). The nasolabial angle was approximately 90°. Intraoral examination revealed bilateral class I molar and canine relationships, associated with marked proclination and protrusion of the maxillary and mandibular incisors (Figures 7D-7F). The mandibular arch exhibited substantial anterior dental crowding, which was more pronounced than that observed in case 1 (Figure 7H). Overall, the clinical findings were consistent with bimaxillary dentoalveolar protrusion associated with lip incompetence, increased facial profile convexity, and mandibular anterior crowding.

**Figure 7:**
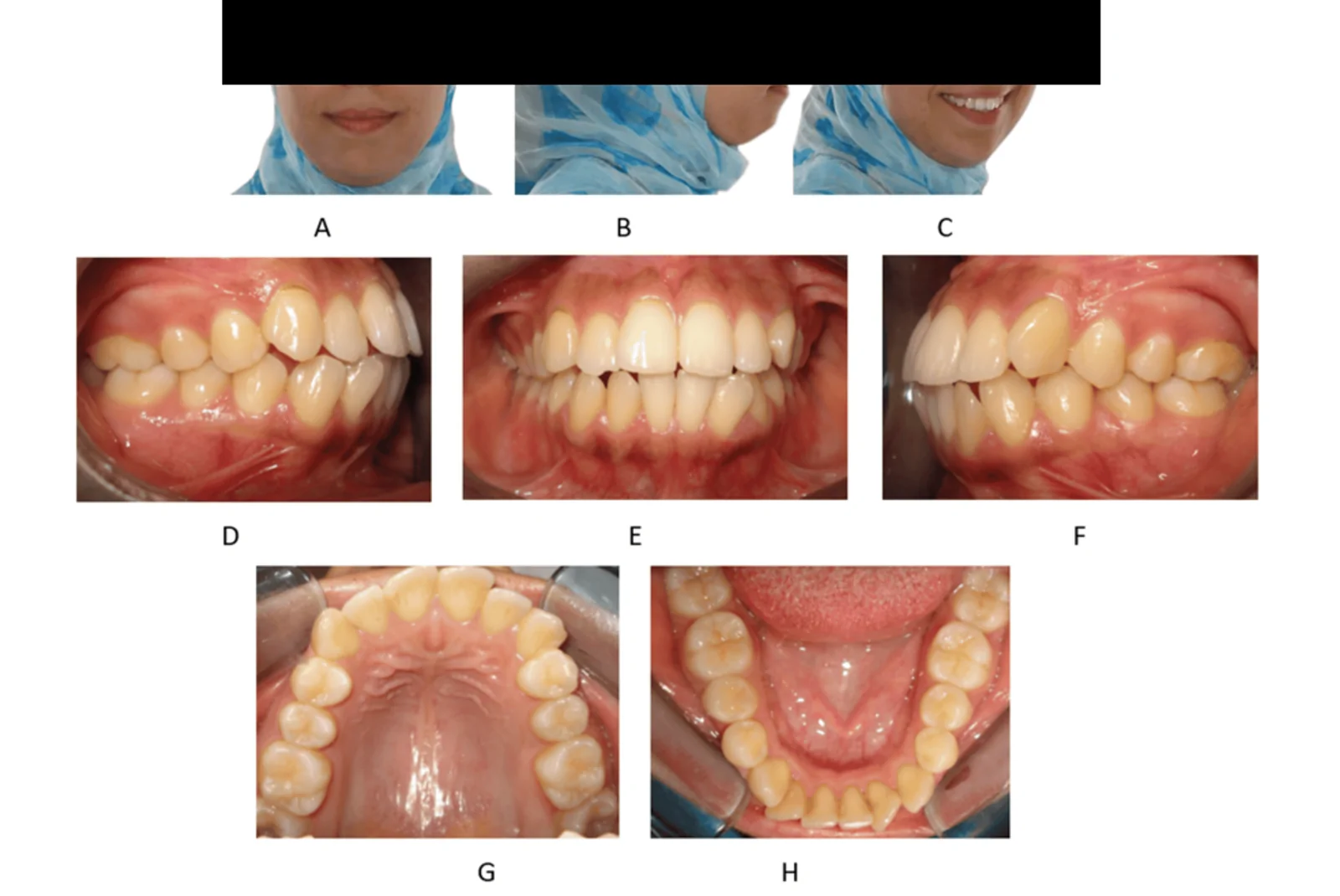
Pretreatment extraoral and intraoral photographs of case 2: frontal facial view (A), profile view (B), three-quarter smiling view (C), right lateral intraoral view (D), frontal intraoral view (E), left lateral intraoral view (F), maxillary occlusal view (G), and mandibular occlusal view (H).

The pretreatment panoramic radiograph (Figure 8A) confirmed a complete permanent dentition, with all four third molars present. No apparent dental or osseous abnormalities were identified within the structures visible on the radiograph. Pretreatment cephalometric analysis (Figure 8B, Table 2) revealed a skeletal class II sagittal relationship, with an ANB angle of 5.0°. Vertical skeletal assessment showed increased SN-GoGn and FMA angles of 42.4° and 36.0°, respectively, consistent with a hyperdivergent skeletal pattern. Dentoalveolar analysis demonstrated marked proclination of the maxillary and mandibular incisors, as reflected by a reduced interincisal angle of 113.7°. Overall, the clinical and cephalometric findings were consistent with a diagnosis of bimaxillary dentoalveolar protrusion associated with a skeletal class II relationship and a hyperdivergent skeletal pattern.

**Figure 8:**
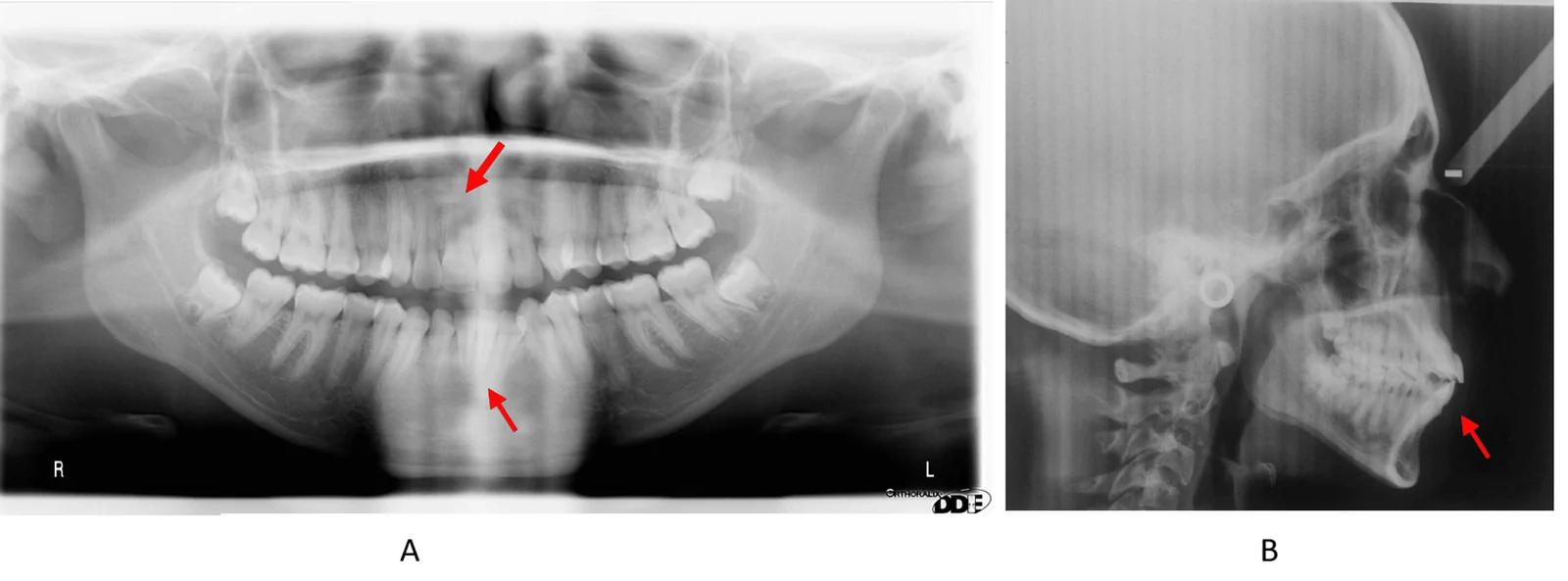
Pretreatment radiographic records. (A) Panoramic radiograph showing the maxillary and mandibular incisor regions (arrows). Incomplete visualization of the mandibular incisor roots is related to their slightly proclined position outside the focal trough. (B) Pretreatment lateral cephalometric radiograph showing marked proclination of the maxillary and mandibular incisors (arrow), consistent with bimaxillary dentoalveolar protrusion.

**Table 2:** SNA, sella–nasion–A point angle; SNB, sella–nasion–B point angle; ANB, A point–nasion–B point angle; AoBo, Wits appraisal (distance between points AO and BO); I to NA, maxillary incisor to nasion–A point line; i to NB, mandibular incisor to nasion–B point line; I to i, interincisal angle; Po to NB, pogonion to nasion–B point line; Occl. to SN, occlusal plane to sella–nasion plane angle; GoGn to SN, mandibular plane (gonion–gnathion) to sella–nasion plane angle; FMA, Frankfort mandibular plane angle; FMIA, Frankfort mandibular incisor angle; IMPA, incisor mandibular plane angle

Parameter	Norm	Pre-treatment	Post-treatment
SNA (˚)	82°	78°	76.0°
SNB (˚)	80°	73.0°	71.0°
ANB (˚)	2°	5.0°	5.0°
AoBo (mm)	−2 mm to + 2 mm	4 mm	3 mm
I to NA (˚)	22°	25.4°	22.9°
I to NA (mm)	4 mm	6.2 mm	5 mm
i to NB (˚)	25°	30.7°	27.8°
i to NB (mm)	4 mm	7.4 mm	5.7 mm
I to i (˚)	131°	113.7°	127.0°
Po to NB (mm)	-	1 mm	1 mm
Occl. to SN (˚)	14°	18°	19.1°
GoGn to SN (˚)	32°	42.4°	41.4°
FMA (˚)	25 ±3°	36.0°	33.0°
FMIA (˚)	67±3°	46.0°	56.0°
IMPA (˚)	88± 3°	98.0°	91.0°

Treatment objectives

The treatment objectives were to address the patient’s esthetic concerns by reducing the bimaxillary dentoalveolar protrusion and improving lip competence and facial profile harmony. Additional objectives included resolving mandibular anterior crowding, maintaining the existing bilateral class I molar and canine relationships, and establishing a stable, functional, and well-intercuspal occlusion.

Treatment plan and progress

An extraction-based orthodontic treatment plan involving the four second premolars was selected to provide the space required for correction of the dentoalveolar discrepancy, resolution of mandibular crowding, and controlled repositioning of the anterior teeth while taking the pretreatment facial profile into consideration.

The decision to extract the second rather than the first premolars was based on the amount and distribution of space required and the planned anteroposterior movements of the anterior and posterior dental segments. In the maxillary arch, only moderate anterior retraction was considered desirable because of the pretreatment soft-tissue profile and nasolabial angle. Extraction of the second premolars was therefore selected to permit space closure through a combination of controlled anterior retraction and mesial movement of the posterior segments rather than maximizing anterior retraction. In the mandibular arch, second premolar extraction provided space for resolution of the anterior crowding and controlled repositioning of the incisors while allowing appropriate management of the posterior segments.

The choice of extraction site was therefore individualized according to the pretreatment dentoalveolar discrepancy, incisor position, anchorage requirements, and facial profile rather than being based on the assumption that second premolar extraction would necessarily produce a smaller soft-tissue response. Previous studies have shown that the extraction pattern influences the distribution of extraction-space closure between anterior retraction and posterior mesial movement; however, substantial individual variability exists, and the resulting soft-tissue response cannot be predicted solely from whether the first or second premolars are extracted [7]. In particular, comparative evidence has shown that changes in lip position and the nasolabial angle may not differ significantly between first- and second-premolar extraction protocols when other treatment-related variables are considered [7].

Following premolar extraction, fixed appliance therapy was initiated with alignment, leveling, and progressive correction of dental rotations in both arches. Canine retraction was subsequently performed, followed by controlled retraction of the incisors and progressive closure of the remaining extraction spaces. Anchorage management was adapted to the planned distribution of space closure between anterior retraction and posterior mesial movement.

During the finishing and detailing phase, 0.019 × 0.025-inch stainless-steel archwires were used to provide three-dimensional control of tooth position, optimize arch coordination, and refine the occlusal relationships (Figures 9A-9C).

**Figure 9:**
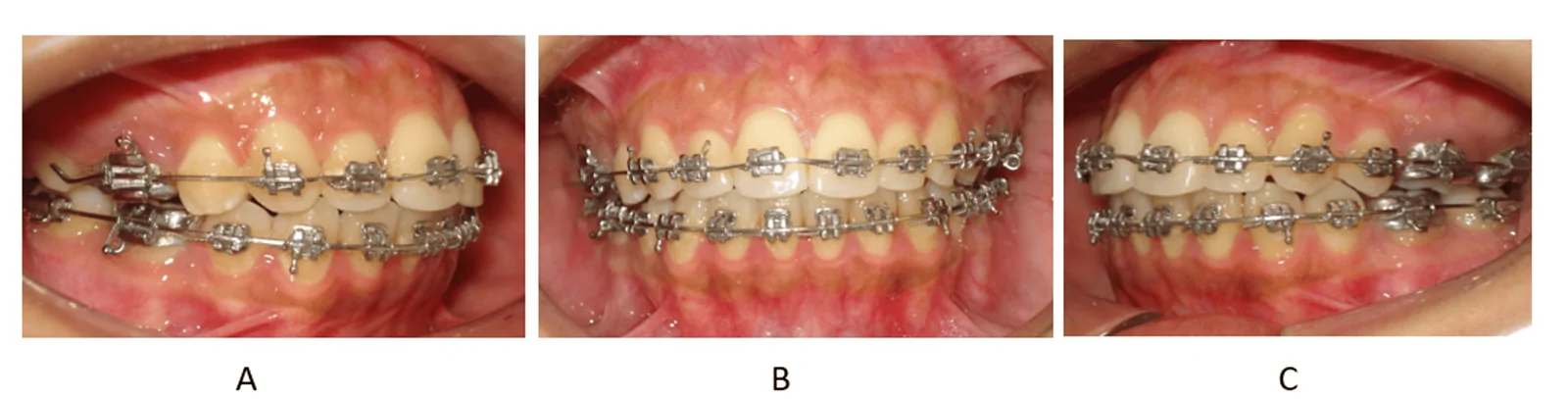
Intraoral photographs obtained during the finishing phase, illustrating refinement of the occlusal relationships and final intercuspation.

Treatment outcomes

After completion of active orthodontic treatment and removal of the fixed appliances, bonded fixed retainers were placed to maintain the achieved tooth positions. In the mandibular arch, the fixed retainer extended from canine to canine, whereas in the maxillary arch, it extended from the right lateral incisor to the left lateral incisor (Figure 10).

**Figure 10:**
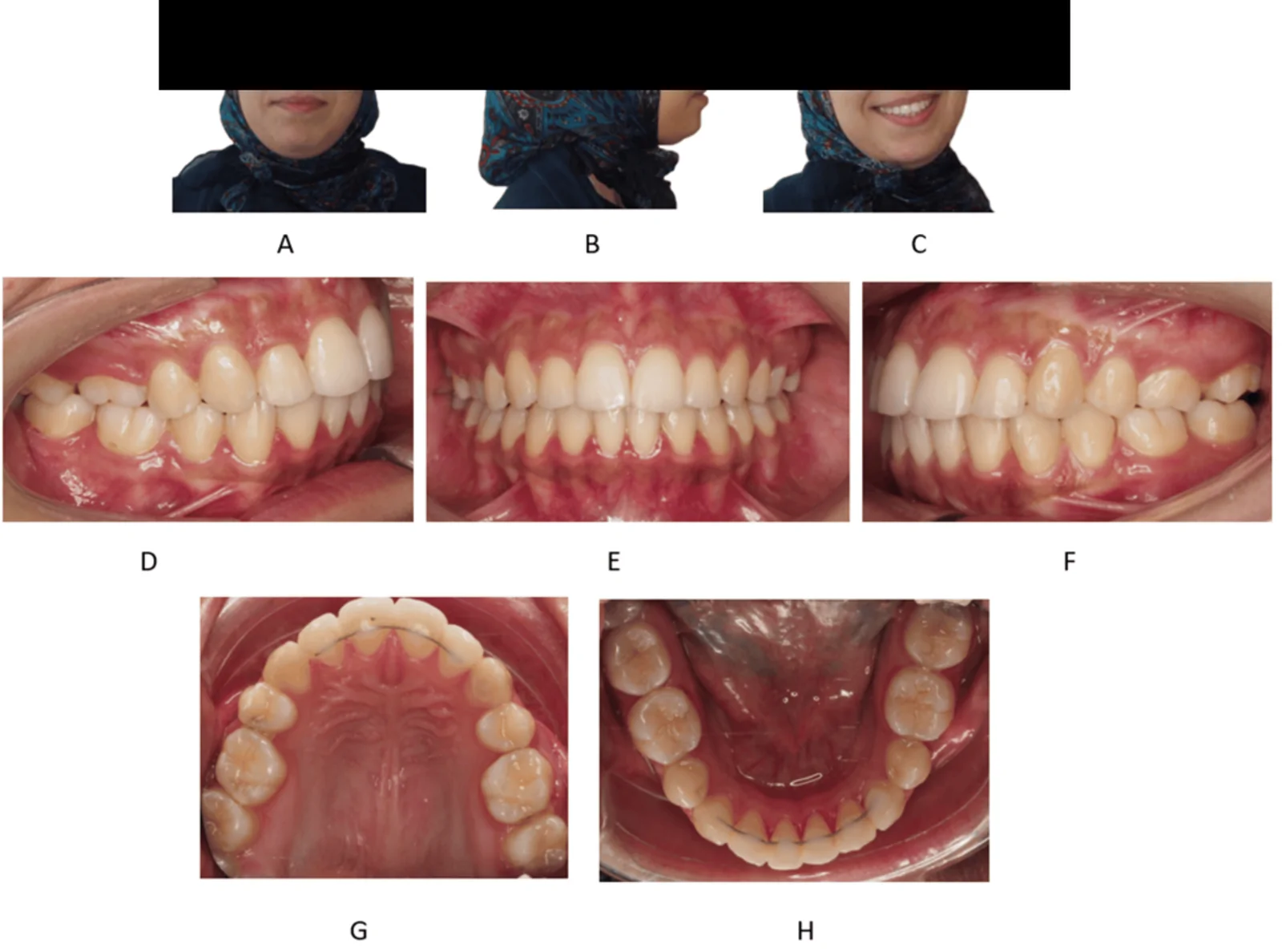
Posttreatment extraoral and intraoral photographs: frontal facial view (A), profile view (B), three-quarter smiling view (C), right lateral intraoral view (D), frontal intraoral view (E), left lateral intraoral view (F), maxillary occlusal view (G), and mandibular occlusal view (H).

The posttreatment panoramic radiograph demonstrated satisfactory root parallelism and overall dental alignment. Regarding the third molars, teeth 28 and 38 remained impacted at the time of follow-up. The lateral cephalometric radiograph showed a clinically apparent improvement in the facial soft-tissue profile, with reduced profile convexity and a more harmonious nose-lip-chin relationship (Figure 11). These soft-tissue changes were assessed qualitatively and were not based on standardized quantitative soft-tissue measurements.

**Figure 11:**
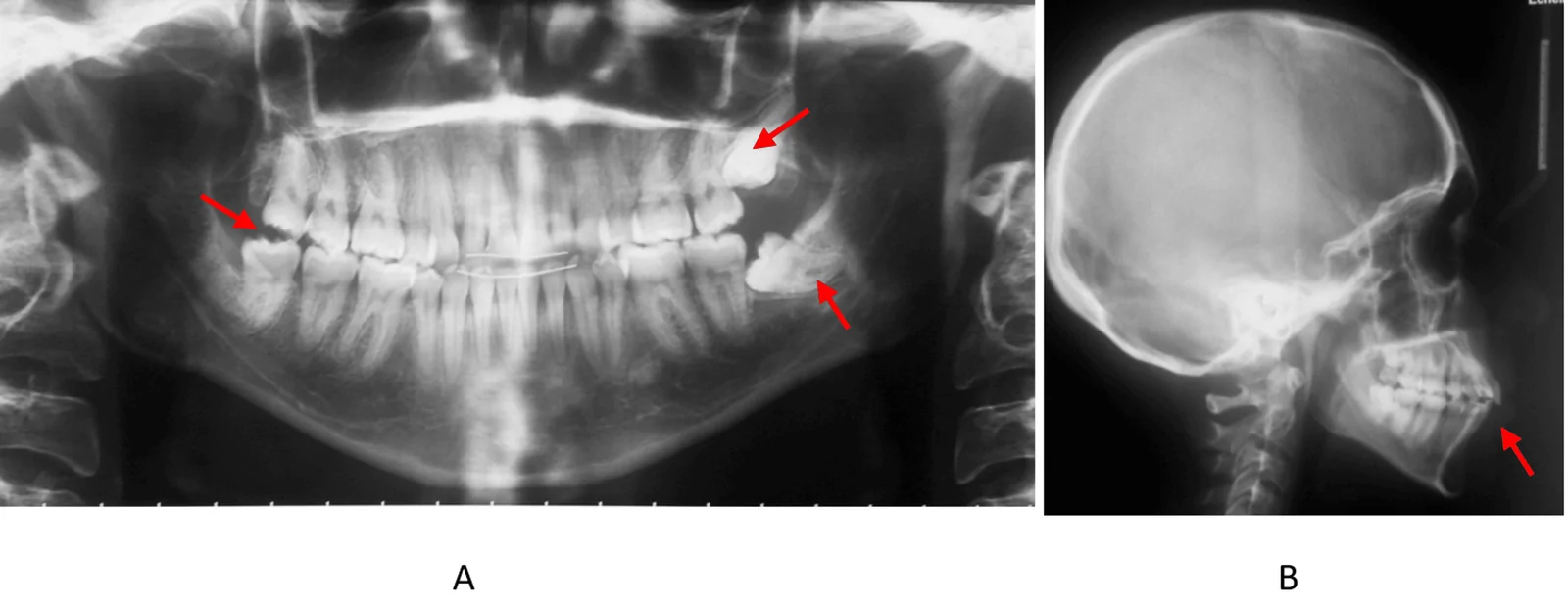
Posttreatment radiographic records. (A) Panoramic radiograph showing teeth 18 and 48 in functional occlusion (arrows), while teeth 28 and 38 remain impacted (arrows). (B) Lateral cephalometric radiograph demonstrating uprighting and improved axial inclination of the maxillary and mandibular incisors following orthodontic treatment (arrows).

Posttreatment cephalometric analysis demonstrated a reduction in maxillary and mandibular incisor proclination. The maxillary incisor inclination relative to NA (U1-NA) decreased from 25.4° to 22.9°, while the mandibular incisor inclination relative to NB (L1-NB) decreased from 30.7° to 27.8°. Accordingly, the interincisal angle increased from 113.7° to 127.0°, reflecting improved axial inclination of the maxillary and mandibular incisors. The IMPA decreased from 98.0° to 91.0°, further indicating uprighting of the mandibular incisors.

In the vertical dimension, the FMA decreased from 36.0° to 33.0°, whereas the ANB angle remained unchanged at 5.0°, indicating maintenance of the sagittal skeletal relationship. The main pretreatment and posttreatment cephalometric measurements are summarized in Table 2.

Pretreatment and posttreatment cephalometric superimposition demonstrated predominantly dentoalveolar and soft-tissue changes, characterized by retraction and uprighting of the maxillary and mandibular incisors. These changes were accompanied by posterior repositioning of the lips, with reduced lip protrusion and an improvement in soft-tissue profile balance (Figure 12).

**Figure 12:**
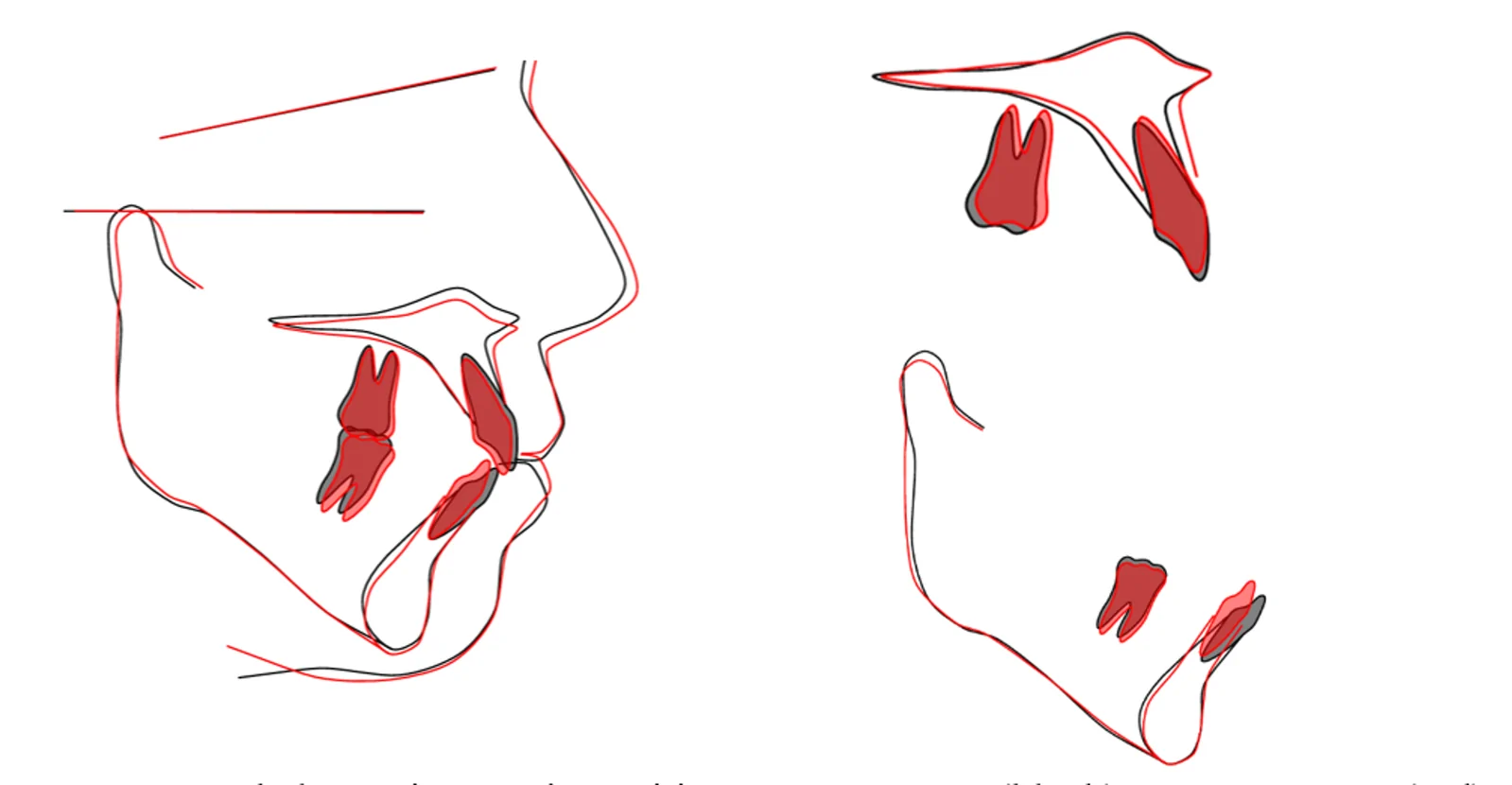
Pretreatment (black) and posttreatment (red) cephalometric superimposition illustrating the skeletal, dentoalveolar, and soft-tissue changes following orthodontic treatment. The image was created using Dental Imaging Studio software (version 1.23; Panoramic Corp).

Both patients presented with bimaxillary dentoalveolar protrusion and were treated with extraction-based fixed orthodontic therapy. In both cases, treatment resulted in incisor retraction and uprighting, with improved lip competence and soft-tissue profile balance.

Qualitative comparison of the pre- and posttreatment clinical profile photographs suggested a more noticeable soft-tissue profile change in case 1, who was treated at 12 years of age during ongoing craniofacial growth (Figure 13A). In case 2, treated at 23 years of age after growth completion, dentoalveolar correction was accompanied by clinically apparent changes in lip position and facial profile, although these changes appeared less noticeable on qualitative photographic assessment (Figure 13B).

**Figure 13:**
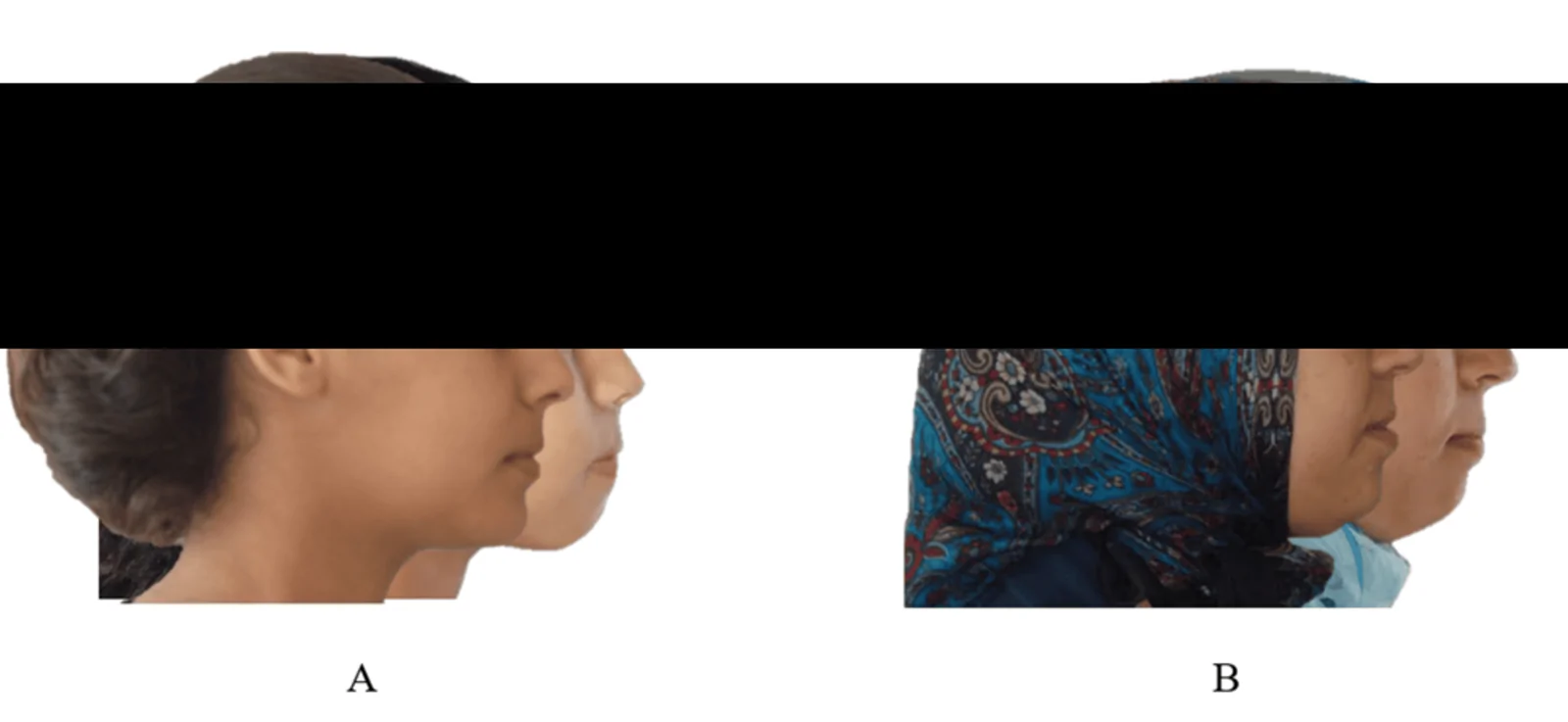
Comparison of the profile view before and after orthodontic treatment in the two patients. A: case 1. B: case 2.

These observations should be interpreted cautiously because no standardized quantitative soft-tissue measurements were performed. Furthermore, the two patients differed not only in age and growth status but also in several baseline skeletal and dental characteristics and underwent different extraction protocols (first versus second premolars). Therefore, the apparent differences in soft-tissue response cannot be attributed specifically to growth status or treatment timing.

## Discussion

This case series describes the dentoalveolar and soft-tissue changes observed in two sisters with a similar clinical presentation of bimaxillary dentoalveolar protrusion who underwent orthodontic treatment at different stages of craniofacial development. In both patients, extraction-based orthodontic treatment resulted in repositioning and reduced proclination of the maxillary and mandibular incisors, accompanied by clinically observed improvements in lip competence and facial profile. Qualitative comparison of the pre- and posttreatment clinical profile photographs suggested a more noticeable soft-tissue profile change in the younger patient. However, this observation should not be interpreted as evidence of a greater treatment response related to growth status, as no standardized quantitative soft-tissue measurements were performed and the two patients differed in their pretreatment dentoskeletal characteristics and extraction protocols.

The changes observed in these two patients are broadly consistent with the available evidence on the treatment of bimaxillary dentoalveolar protrusion. The extraction pattern itself may influence the distribution of space closure and should therefore be considered when interpreting dentoalveolar and facial profile changes [9]. Clinical studies and systematic reviews have shown that premolar extraction followed by anterior tooth retraction is generally associated with lip retraction and changes in the nasolabial angle, although the magnitude of these changes varies considerably among patients [10,11]. The relationship between incisor position and soft-tissue characteristics is particularly relevant in patients with bimaxillary protrusion, in whom variations in skeletal pattern may also be associated with differences in incisor and facial soft-tissue characteristics [12]. In a prospective study involving patients with class I bimaxillary protrusion, extraction-based orthodontic treatment resulted in retraction of both the incisors and lips, with an association between incisor movement and changes in the soft-tissue profile [13]. Similarly, a retrospective study of patients with bimaxillary protrusion reported cephalometric changes following premolar extraction treatment [14].

In the present case series, the cephalometric changes were consistent with these previously reported dentoalveolar trends. In the younger sister, U1-NA decreased from 24.7° to 22.0° and L1-NB from 30.3° to 26.8°. In the older sister, U1-NA decreased from 25.4° to 22.9° and L1-NB from 30.7° to 27.8°. In both patients, reduced incisor proclination was accompanied by clinically apparent changes in lip competence and facial profile. However, these soft-tissue changes were assessed clinically and photographically rather than through standardized quantitative measurements and should therefore be interpreted qualitatively.

The relationship between incisor movement and soft-tissue response is neither linear nor strictly proportional. Several studies have demonstrated variability in facial and lip morphology following extraction-based orthodontic treatment [15-17]. This variability may reflect differences in baseline soft-tissue characteristics, the magnitude and type of tooth movement, and other patient- and treatment-related factors.

Control of incisor inclination is also an important biomechanical consideration during anterior retraction following premolar extraction. Appropriate control of incisor inclination and torque during space closure allows better management of anterior tooth position and helps minimize uncontrolled lingual crown tipping [8,18]. Changes in incisor position may, in turn, contribute to the soft-tissue profile changes observed following extraction-based orthodontic treatment of bimaxillary dentoalveolar protrusion [13]. Nevertheless, the extent of the soft-tissue response cannot be predicted from incisor movement alone.

More recent evidence further supports the influence of individual skeletal and soft-tissue characteristics on treatment response. Erdem et al. investigated lip changes following premolar extraction in patients with high and normal vertical growth patterns, highlighting the potential relevance of baseline vertical skeletal characteristics when interpreting soft-tissue response [19]. Qadeer et al. evaluated the influence of lip thickness and lip competence on soft-tissue changes following orthodontic treatment [20]. Taken together, these findings emphasize the multifactorial nature of the soft-tissue response and the difficulty of predicting changes in lip position solely from the magnitude of incisor retraction [19,20].

In the younger patient, dentoalveolar correction occurred concurrently with changes in sagittal and vertical skeletal parameters. The ANB angle decreased from 7.5° to 6.1°, SN-GoGn from 37.5° to 34.1°, and FMA from 29.3° to 26.3°. Because this patient was undergoing active craniofacial growth during treatment, these skeletal changes cannot be attributed exclusively to orthodontic treatment. Physiological craniofacial growth and maturation may have contributed to the observed changes in skeletal relationships and facial profile [6]. Furthermore, baseline vertical skeletal pattern may influence the soft-tissue response to premolar extraction [19]. Accordingly, the posttreatment findings in the younger patient should be interpreted as reflecting the combined influence of orthodontic treatment, ongoing craniofacial development, baseline dentoskeletal characteristics, and individual soft-tissue factors rather than as evidence of a specific growth-related treatment effect.

An important limitation of the present comparison is that the two patients did not undergo identical extraction protocols. The younger patient underwent extraction of the four first premolars, whereas the older patient underwent extraction of the four second premolars. The extraction site may influence the distribution of extraction-space closure between anterior retraction and posterior mesial movement and therefore represents a potential confounding factor when comparing dentoalveolar and soft-tissue changes between the two cases [9]. Thus, differences in extraction protocol should be considered together with anchorage requirements, the amount and type of anterior tooth movement, and individual soft-tissue characteristics when interpreting the outcomes of the two patients.

The patients also differed in their pretreatment dentoskeletal characteristics, particularly in their sagittal and vertical skeletal relationships and in the severity of mandibular crowding. These baseline differences are clinically relevant because vertical skeletal pattern [19] and individual soft-tissue characteristics such as lip thickness and competence [20] may influence the response to extraction-based orthodontic treatment. Consequently, although qualitative comparison of the clinical profile photographs suggested a more noticeable soft-tissue profile change in the younger patient, the present case series cannot determine whether this apparent difference was related to growth status, baseline dentoskeletal characteristics, extraction pattern, treatment mechanics, individual soft-tissue characteristics, or a combination of these factors. The comparison should therefore be regarded as descriptive and hypothesis-generating rather than as evidence of an effect of treatment timing.

The intrafamilial nature of these cases provides an interesting clinical perspective by allowing treatment responses to be described in two closely related individuals. However, familial relatedness does not eliminate genetic, developmental, environmental, or individual biological variability and does not compensate for differences in pretreatment characteristics and treatment protocols. It should therefore be regarded as a distinctive descriptive feature of this case series rather than as a means of controlling biological variability.

Several limitations should therefore be acknowledged. First, this report includes only two patients and does not permit statistical comparison or causal inference. Second, the patients differed in age, growth status, baseline dentoskeletal characteristics, severity of crowding, and extraction protocol. Third, soft-tissue changes were evaluated qualitatively from clinical photographs and cephalometric records rather than through standardized quantitative soft-tissue measurements. Future studies using larger samples, standardized quantitative soft-tissue measurements, comparable treatment protocols, and longitudinal follow-up are needed to better clarify the respective contributions of growth, orthodontic mechanics, and individual biological characteristics to the soft-tissue response following extraction treatment.

## Conclusions

This case series illustrates the dentoalveolar and soft-tissue changes following extraction-based orthodontic treatment of bimaxillary dentoalveolar protrusion in two sisters treated at different stages of craniofacial development. In both patients, incisor retraction and uprighting were accompanied by improved lip competence and facial profile balance. The younger patient, treated during active craniofacial growth, exhibited more pronounced soft-tissue profile changes, whereas the changes observed in the adult patient were predominantly dentoalveolar.

Although these findings raise the possibility that growth status may contribute to differences in dentofacial response, no causal relationship can be established because of the limited number of cases and differences in baseline characteristics and extraction protocols. Larger prospective comparative studies are needed to clarify the respective contributions of growth status, treatment timing, incisor repositioning, and extraction strategy to dentoalveolar and soft-tissue outcomes.
